# A *de novo* 1.6Mb microdeletion at 19q13.2 in a boy with Diamond-Blackfan anemia, global developmental delay and multiple congenital anomalies

**DOI:** 10.1186/s13039-016-0268-2

**Published:** 2016-08-02

**Authors:** Haiming Yuan, Zhe Meng, Liping Liu, Xiaoyan Deng, Xizi Hu, Liyang Liang

**Affiliations:** 1 Sun Yat-Sen Memorial Hospital, Sun Yat-Sen University, Guangzhou, 510120 Guangdong China; 2 Guangzhou kingmed center for clinical laboratory Co.,Ltd., Guangzhou, 510005 Guangdong China; 3 KingMed School of Laboratory Medicine Guangzhou Medical University, Guangzhou, 510005 Guangdong China; 4 Wuhan women and children medical healthcare center, Department of Obstetrics and Gynecology, Wuhan, 430016 Hubei China; 5 Fairmont Preparatory Academy, California, 92801 USA

**Keywords:** Microdeletion, 19q13.2, Diamond-Blackfan Anemia, Global developmental delay, Cognitive impairments, Behavior problems

## Abstract

**Backgroud:**

Microdeletions at 19q13.2 are very rare. Only two cases have been previously described. Here we report a 2-year-2-month old boy with Diamond-Blackfan anemia, global developmental delay, cognitive impairments, distinctive facial features, behavior problems, skeletal and genital dysplasia.

**Case presentation:**

A *de novo* 1.6 Mb microdeletion at 19q13.2q13.31 was detected by chromosomal microarray analysis. Haploinsufficiency of the *RPS19* gene is known to cause Diamond-Blackfan anemia, other features in this patient are likely due to the deletion of other candidate genes such as *PAFAH1B3*, *ERF*, *LIPE* and *GSK3A*.

**Conclusion:**

The deletion detected in our patient overlapped and was significantly smaller than the ones previously reported, which offered the opportunity to further define the critical region for this proposed contiguous gene deletion syndrome.

**Electronic supplementary material:**

The online version of this article (doi:10.1186/s13039-016-0268-2) contains supplementary material, which is available to authorized users.

## Background

Microdeletions at 19q13.2 have rarely been reported. Cario [1] and Tentler [2] each reported a case with microdeletion at 19q13.2 defined by FISH analysis respectively, thus the sizes and boundaries of the deletions were not precisely delineated. The clinical features of the two patients included Diamond Blackfan Anemia (DBA), global developmental delay, mental retardation, distinctive facial features and skeletal malformations [1, 2]. Haploinsufficiency of the *RPS19* (OMIM 603474) gene involved in the deletions is known to cause DBA. DBA is a pure red-cell hypoplasia characterized by defective erythroid progenitor maturation and normal numbers and function of other haemopoietic cells [3–5]. It has been observed that patients with 19q13.2 microdeletion involving the *RPS19* gene presented with a more complex clinical phenotype than those caused only by sequence variants in *RPS19* gene [1–7]. Tentler et al. proposed that 19q13.2 deletion represented a novel contiguous gene deletion syndrome [2]. Here, we report a *de novo* 1.6 Mb microdeletion at 19q13.2q13.31 detected by chromosomal microarray in a 2-year-2-month old boy with many common features as reported in previous cases. The deletion detected in our patient was smaller and better defined by high resolution chromosomal microarray analysis. This case offered the opportunity for defining the critical region and discussing candidate genes associated with different phenotypes.

## Case presentation

The proband was the first child of healthy unrelated parents and family history was unremarkable. Intrauterine growth retardation and oligohydramnios was noticed by ultrasound examination at 8 months of pregnancy. Because of progressive intrauterine growth retardation (IUGR), a delivery by cesarean section was performed at 37 weeks of gestation. Birth weight was 3.1 kg, length 47 cm (<−2SD) and head circumference 34 cm. Apgar scores were all 8. Feeding difficulty was noted at all times. At the age of 9 months he was referred to a pediatric clinic because of pallor. Hemoglobin concentration was 64 g/l and no reticulocytes were detected in peripheral blood. Bone marrow aspirate showed a selective decrease in erythroid precursors but otherwise normal cellularity. Hemoglobin concentration was recovered from 64 to 106 g/l after corticosteroid treatment.

**Fig. 1:**
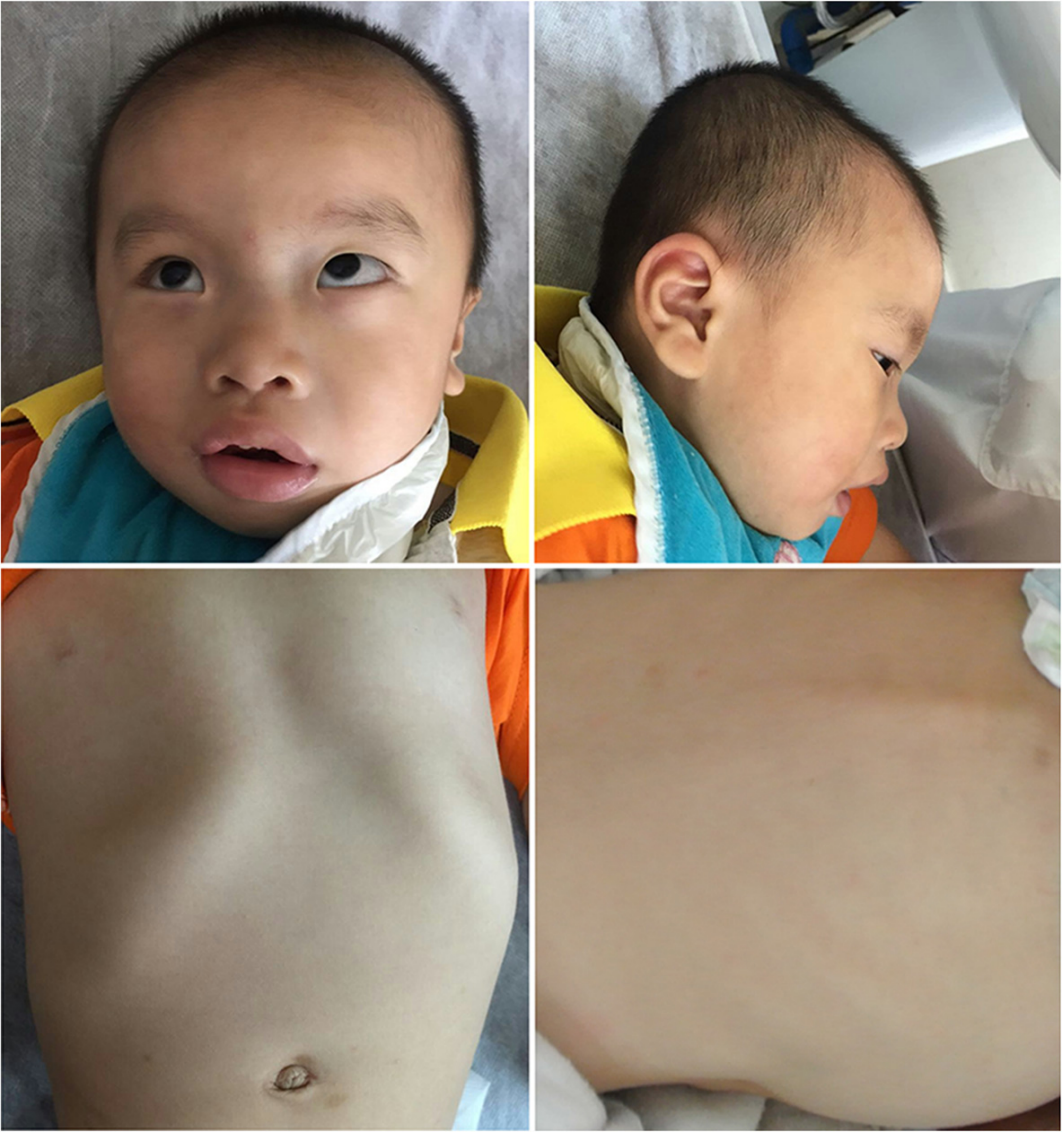
The proband at 2-year-2-month age. Note cranial deformities, mild craniosynostosis, broad forehead, auricle dysplasia, hypertelorism, strabismus, broad nose with depressed nasal bridge, thick lips, micrognathia and open-mouthed expression, rib protrusion and kyphosis

The proband was 2 years 2 months old at the time of molecular evaluation. His weight was 9.9 kg (<−2SD), height 78 cm (<−3SD), and head circumference 47.5 cm, which indicated persistent failure to thrive. The developmental milestones were delayed: he raised his head at 7 months, sat alone at 1 year and could not independently walk yet. Language development was significantly delayed and he had almost complete absence of speech. He had moderate cognitive impairments. His distinctive facial features were characterized by cranial deformities, mild craniosynostosis, broad forehead, auricle dysplasia, arched and sparse eyebrows, hypertelorism, nystagmus and strabismus, broad nose with depressed nasal bridge, thick lips, teeth dysplasia, micrognathia, open-mouthed expression and drooling. He had skeletal abnormality including rib protrusion and kyphosis, but with normal level of calcium, phosphorus and alkaline phosphatase (Fig. 1). His abnormal behavior included mild self-mutilation, fingers biting, tongue stretching, anxiety and hyperactivity. He was insensitive to pain. Severe hypotonia and sleep disorders were present. Micropenis, small testes and anal fissure were detected. He had gastrointestinal dysfunction and suffered from frequent diarrhea. He would have high body temperature at night and return to normal at daylight spontaneously. Brain MRI, ultrasound and X-ray examinations for heart and lungs were all normal.

## Methods

### Chromosome karyotype analysis

Cytogenetic investigations (GTG banding) on 20 metaphases obtained from PHA-stimulated peripheral lymphocytes of the patient were performed following standard protocols.

### Chromosomal microarray analysis

Chromosomal microarray analysis was performed for the patient and both parents by Affymetrix Cytoscan HD Array (Affymetrix, USA). Genomic DNA was extracted from peripheral blood using a commercial kit (Qiagen). The labeling and hybridization procedures were performed following manufacturer’s instructions. The raw data of chromosomal microarray was analyzed by Affymetrix Chromosome Analysis Suite Software.

### Confirmation of 19q13.2q13.31deletion

The deletion was further confirmed using quantitative real-time PCR analysis. Primer sequences and descriptions were included in Additional file 1: Table S1.

## Results

**Fig. 2:**
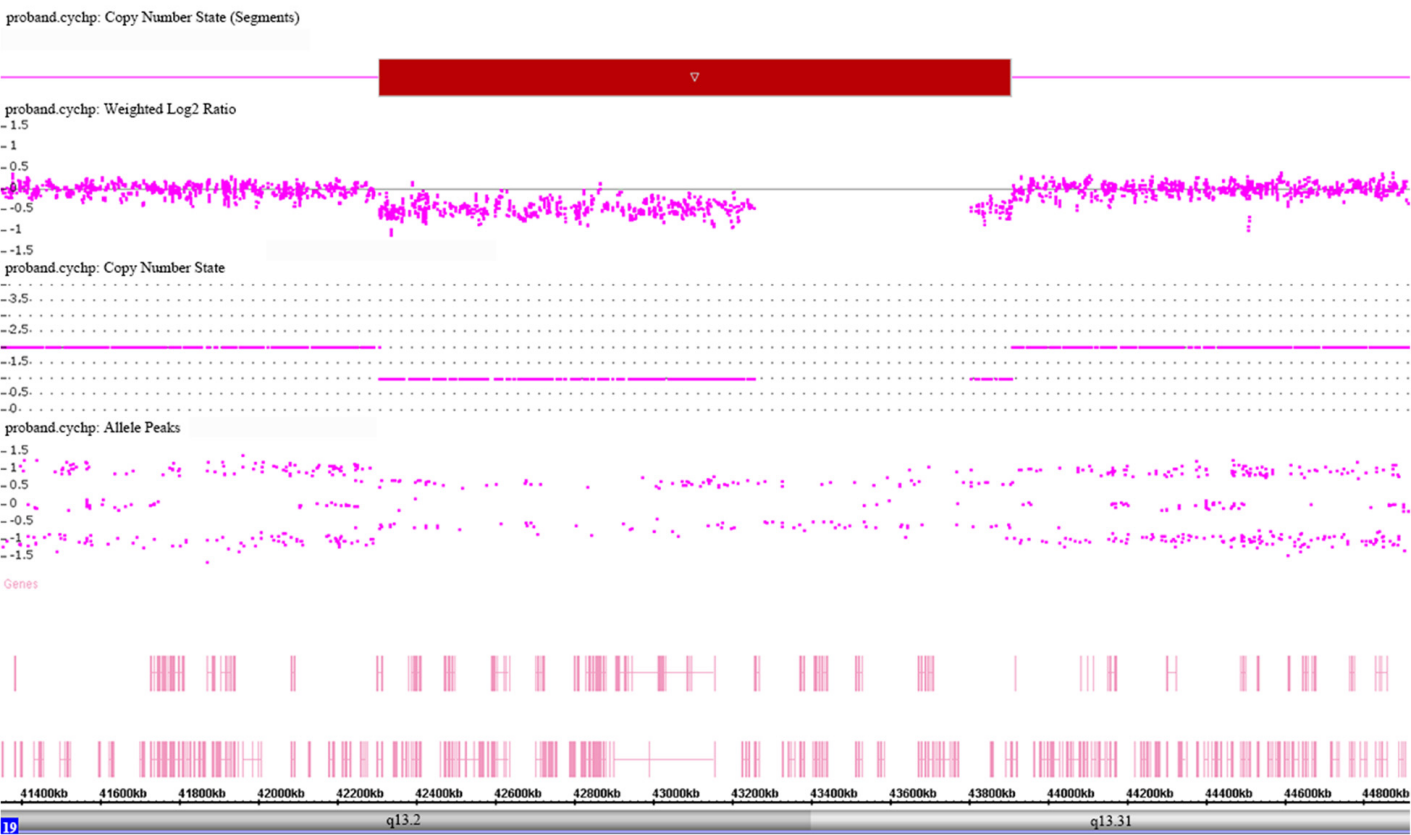
Affymetrix cytoscan HD array analysis including weighted log2 ratio (upper), copy number state (middle) and allele peaks (lower) are shown for chromosome 19. The result shows microdeletion at 19q13.2q13.31. The genomic coordinates (hg19): chr19:42,306,042-43,906,653. The microdeletion region is denoted by a red bar

Standard chromosome analysis of peripheral blood by GTG banding was normal (data not shown). A 1.6 Mb microdeletion at 19q13.2q13.31 (chr19:42,306,042-43,906,653) was detected by chromosomal microarray analysis (Fig. 2). Parental chromosomal microarray analysis were normal. Thus, the proband carried a *de novo* copy number variant. The deletion was further confirmed by quantitative real-time PCR analysis (data not shown).

## Discussion and conclusion

**Fig. 3:**
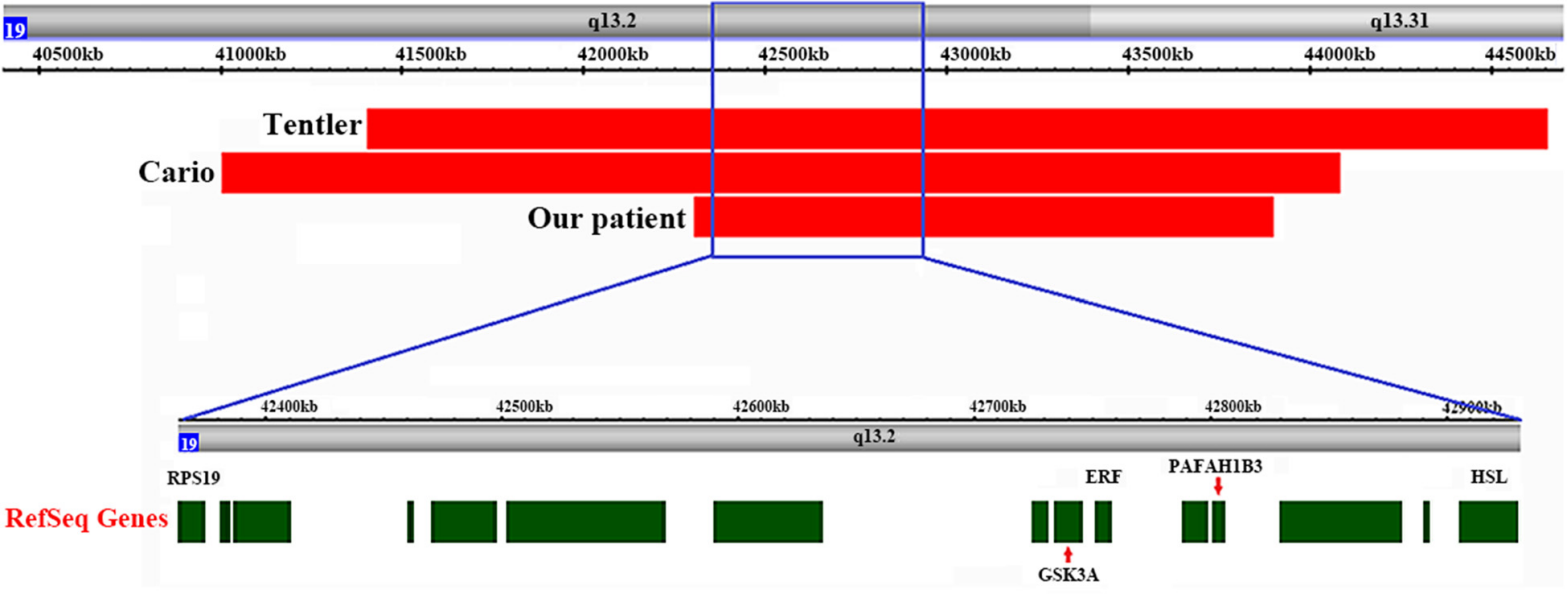
Top panel shows a genome view of all deletion cases (red colored custom tracks) relative to the genomic coordinates at 19q13.2q13.31 region, extracted from Human Genome Build 37 (hg19). Blue block diagram represents critical region of 19q13.2. Bottom panel shows the zoomed-in 19q13.2 critical region encompassing candidate genes

Microdeletions at 19q13.2 are very rare. So far, only two cases carrying a microdeletion at 19q13.2 have been reported who share similar clinical features including Diamond-Blackfan anemia, global developmental delay, skeletal abnormalities and intellectual disability. Both cases were detected by FISH analysis and the relative breakpoints and sizes estimation of the deletions were determined, whereas candidate genes except for *RPS19* gene in this interval responsible for the complex clinical features of the patients were not identified [1, 2]. Our patient reported here carries a *de novo* 1.6 Mb microdeletion at 19q13.2q13.31 uncovered by high resolution chromosomal microarray analysis and no other clinical significant copy number variants are detected. The positions and sizes of three cases are delineated in Fig. 3. The clinical presentations in all three cases are summarized in Table 1.

**Table 1:** Clinical features observed in patients with 19q13.2 deletion

Phenotypic characteristic	Our patient	Cario et al.	Tentler et al.
Sex	Male	Male	Male
Age	2 years 2 months	13 months	12 years
Size of the deletion (Mb)	1.6 Mb	about 3.0 Mb	about 3.2 Mb
Genomic location	chr19:42306042-43906653	19q13.2q13.31	19q13.2q13.31
Methods	microarray	interphase FISH	fibre-FISH
Diamond-Blackfan Anemia	+	+	+
Feeding difficulties	+	NR	NR
IUGR	+	NR	NR
Global developmental delay			
Growth delay (short stature)	+	+	+
Delayed motor development	+	+	+
Language delay	+	+	+
Cognitive impairments	+	+	+
Craniofacial features			
Macrocephaly	-	+	+
Cranial deformities	+	+	+
Broad forehead	+	+	NR
Auricle dysplasia	+	+	NR
Hypertelorism	+	+	NR
strabismus	+	+	NR
Broad nose with depressed nasal bridge	+	+	NR
Thick lips	+	+	NR
Teeth dysplasia	+	NR	NR
Open-mouthed expression	+	+	NR
Drooling	+	+	NR
Skeletal abnormalities	+	+	+
Genital anomalies	+ (small testes)	+ (cryptorchidism)	NR
Hypotonia	+	+	NR
Behavior problems	+	+	NR
Body temperature dysregulation	+	NR	NR

*IUGR* intrauterine growth retardation, *NR* not reported

Haploinsufficiency of the *RPS19* gene is responsible for the Diamond-Blackfan anemia phenotype in these patients. *RPS19* deletion is not likely to cause other features observed in these individuals since none of the patients with DBA caused by *RPS19* gene point mutations has developmental delay, intellectual disability or dysmorphism features [5–7]. Furthermore, a girl reported to carry a *de novo* balanced translocation t(X;19) (p21;q13) which interrupted the *RPS19* gene also had normal development without skeletal malformations [3, 4]. These findings further suggested that other genes at 19q13.2 locus contributed to other clinical features observed in these patients.

Based on this notion, we analyzed all genes involved in the deleted interval detected in our patient in Additional file 2: Table S2. And we identified several candidate genes that could explain the additional features seen in our patient. The *PAFAH1B3* (OMIM 603074) gene maps in 19q13.2 region. Mutations or deletions of *PAFAH1B1* gene result in Miller-Dieker syndrome characterized by lissencephaly, severe intellectual disability, developmental delay, distinctive facial features, seizures, hypotonia and feeding difficulties. PAFAH1B1 is a subunit of a brain platelet-activating factor acetylhydrolase (PAFAH1B) where it forms a heterotrimeric complex with two hydrolase subunits, referred to as 29 kDa (PAFAH1B3) and 30 kDa (PAFAH1B2). In the brain, PAFAH1B complex regulates the level of platelet activating factor, which is thought to be involved in neuronal migration essential for normal brain development and function [8–10].

Haploinsufficiency of *ERF* (OMIM 611888) gene involved in this interval leads to complex craniosynostosis recognized by multiple-suture synostosis, craniofacial dysmorphism, Chiari malformation, behavior problems and language delay [11, 12].

Among other genes in this interval, we identified the *LIPE* (OMIM 151750) and *GSK3A* (OMIM 606784) as candidate genes. Animal model shows that hormone sensitive lipase encoded by *LIPE* gene is a multifunctional fatty acyl esterase that causes male infertility and decreased testes weight and also plays an important role in the function of adipocytes, pancreatic-cells, and adrenal cortical cells [13–15]. Glycogen synthase kinase 3 (GSK3), encoded by *GSK3A* and *GSK3B* genes, is shown to be expressed ubiquitously in all mammalian tissues and is also essential for normal sperm function required for male fertility [16, 17]. Furthermore GSK3 is implicated to be associated with a variety of diseases including mood disorders, Alzheimer disease, diabetes and cancer, and plays a role in embryo development as well [17–19]. Taken together, these candidate genes involved in 19q13.2 region could explain the complex clinical phenotype observed in our patient.

In conclusion, we report a *de novo* microdeletion at 19q13.2q13.31 in a patient with DBA, global developmental delay, cognitive impairments, facial dysmorphism, behavior problems, skeletal and genital dysplasia. Our patient exhibited strikingly similar clinical phenotypes among those patients with 19q13.2 microdeletions. Haploinsufficiency of the *RPS19* is the cause of DBA and several candidate genes responsible for other clinical features are identified in this interval, which further suggests a novel contiguous gene deletion syndrome and the critical region at 19q13.2 locus is defined as well.

## Abbreviations

*DBA* Diamond Blackfan Anaemia; *GSK3* glycogen synthase kinase 3; *IUGR* intrauterine growth retardation

## Additional files

Additional file 1: Table S1. Primers information. (DOCX 23 kb) Additional file 2: Table S2. All genes in the deleted interval detected in our patient. (DOCX 19 kb)
