# Supplementary material for: A de novo 1.6Mb microdeletion at 19q13.2 in a boy with Diamond-Blackfan anemia, global developmental delay and multiple congenital anomalies
Source: Mol Cytogenet. 2016 Aug 2;9:58. doi: 10.1186/s13039-016-0268-2 (PMC4970238; doi:10.1186/s13039-016-0268-2)
Supplement: Additional file 1: Table S1. — Primers information. (DOCX 23 kb) [file 13039_2016_268_MOESM1_ESM.docx]

**Methods:** Quantitative real-time polymerase chain reaction (qPCR) technology using SYBR Green I reagents was performed for confirmation of CMA results. Genomic DNA was extracted from peripheral blood using a commercial kit (Qiagen). RPS19 and CD177 genes at 19q13.2q13.31 interval were selected for qPCR confirmation. Normal human genomic DNA was used as reference DNA for qPCR testing. The quality and quantity of DNA samples were assessed using a NanoDrop ND-1000 UV-VIS spectrophotometer (NanoDrop Technologies, Wilmington, DE) and agarose gel electrophoresis. Test primers targeted to DNA sequences within deleted interval and reference primers were designed. (Supplementary Table 1).

Table S1: Primers information

| Primer name Primer sequences (5’-3’) product length (bp) |
| --- |
| Primer set: Test primers  RPS19 F：5' CGTGCTTTTCCCACTGTTTT '3 102  R：5' CGTAGGGAGCAAGCTCTTTG '3  CD177 F：5' CAGCATGTGTGGAAGGTGTC '3 100  R：5' CAATGAGCATCAACGTGTCC '3  Primer set: Reference primer  ACTB F：5' TCGTGCGTGACATTAAGGAG '3 110  R：5' GTCAGGCAGCTCGTAGCTCT '3 |

When preparing assays for CNV confirmation, there are four groups of reactions: (1) DNA plus test primers, (2) DNA plus reference primers, (3) reference DNA plus test primers, and (4) reference DNA plus reference primers. Each reaction was analyzed in triplicate for both DNA samples (test and reference samples) and both sets of primers (test and reference primers). qPCRs were performed in a 20µl volume including 25ng template DNA, 200nM of each primer, and 1×Premix-Choice with ROX reference dye in an initial denaturation of 93℃ for 10 min, followed by 35cycles of 93℃for 1 min and 60℃ for 1 min, and 72℃ for 1 min. Dissociation curve and agarose gel electrophoresis were used to evaluate the specificity and efficiency for each set of test primers.

qPCR data normalization, calculation, and interpretation

The comparative threshold cycle (C_t_) method of relative quantitation was selected to calculate the relative DNA copy numbers in test samples. Results were expressed in terms of the C_t_ value at which the fluorescence intensity for the SYBR green I dye with ROX as the passive reference dye exceeds the detection threshold. The formula used for the copy number calculation is 2×2^ΔΔCt^ (the actual copy numbers of test gene in test samples), where ΔΔC_t_=ΔC_t_ (reference sample)-ΔC_t_ (test sample). ΔC_t_ represents the difference between the C_t_ of a test gene and the C_t_ of the selected reference gene. Cut-off values for deletion were set arbitrarily with ΔΔC_t_= -1±0.35 (1×35%) for a deletion (e.g., based on the formula, the accepted actual copy number for a deletion it should be 1±0.35).

**Results:**

qPCR data show one copy of the test genes RPS19 and CD177 in test DNA and two copies of the two genes in reference DNA. The result of qPCR is consistence with the one of CMA.

Supplementary Table 2: qPCR data of RPS19 gene

| Test sample | C_t_ | C_t_(average) | Reference sample | C_t_ | C_t_(average) |
| --- | --- | --- | --- | --- | --- |
| RPS19 (1) | 23.381 | 23.377 | RPS19 (1) | 22.343 | 22.333 |
| RPS19 (2) | 23.359 |  | RPS19 (2) | 22.348 |  |
| RPS19 (3) | 23.391 |  | RPS19 (3) | 22.308 |  |
| ACTB (1) | 21.684 | 21.83 | ACTB (1) | 21.848 | 21.902 |
| ACTB (2) | 21.873 |  | ACTB (2) | 21.856 |  |
| ACTB (3) | 21.934 |  | ACTB (3) | 22.002 |  |

△Ct (reference sample)＝22.333-21.902=0.431

△Ct (test sample)＝23.377-21.83=1.547

△△Ct=0.431-1.547=-1.116

2×2^△△Ct^=2×2^-1.116^=0.923

Supplementary Table 3: qPCR data of CD177 gene

| Test sample | C_t_ | C_t_(average) | Reference sample | C_t_ | C_t_(average) |
| --- | --- | --- | --- | --- | --- |
| CD177 (1) | 23.904 | 24.164 | CD177 (1) | 22.762 | 22.731 |
| CD177 (2) | 24.274 |  | CD177 (2) | 22.733 |  |
| CD177 (3) | 24.315 |  | CD177 (3) | 22.699 |  |
| ACTB(1) | 22.485 | 22.699 | ACTB(1) | 22.415 | 22.522 |
| ACTB(2) | 22.626 |  | ACTB(2) | 22.576 |  |
| ACTB(3) | 22.985 |  | ACTB(3) | 22.575 |  |

△Ct (reference sample)＝22.731-22.522=0.209

△Ct (test sample)＝24.164-22.699=1.465

△△Ct=0.209-1.465=-1.256

2×2^△△Ct^=2×2^-1.256^=0.838
