# Supplementary material for: A de novo 1.6Mb microdeletion at 19q13.2 in a boy with Diamond-Blackfan anemia, global developmental delay and multiple congenital anomalies
Source: Mol Cytogenet. 2016 Aug 2;9:58. doi: 10.1186/s13039-016-0268-2 (PMC4970238; doi:10.1186/s13039-016-0268-2)
Supplement: Additional file 2: Table S2. — All genes in the deleted interval detected in our patient. (DOCX 19 kb) [file 13039_2016_268_MOESM2_ESM.docx]

**Table S2: All genes in the deleted interval detected in our patient**

| gene | Chromosome location | Genomic location (hg19) | annotation |
| --- | --- | --- | --- |
| CEACAM3 | 19q13.2 | 42300522~42315591 | A member of the family of carcinoembryonic antigen-related cell adhesion molecules (CEACAMs). Diseases associated with CEACAM3 include extragonadal germ cell tumor and sinonasal undifferentiated carcinoma. |
| LYPD4 | 19q13.2 | 42339356~42348774 | Not described |
| DMRTC2 | 19q13.2 | 42348806~42356398 | Not described |
| RPS19 | 19q13.2 | 42363988~42375484 | Refer to discussion section |
| CD79A | 19q13.2 | 42381190~42385439 | This gene encodes the Ig-alpha protein of the B-cell antigen component. |
| ARHGEF1 | 19q13.2 | 42387240~42411604 | The encoded protein may form complex with G proteins and stimulate Rho-dependent signals. |
| RABAC1 | 19q13.2 | 42460833~42463530 | Not described |
| ATP1A3 | 19q13.2 | 42470628~42498428 | The protein encoded by this gene belongs to the family of P-type cation transport ATPases, and to the subfamily of Na+/K+ -ATPases. Na+/K+ -ATPase is an integral membrane protein responsible for establishing and maintaining the electrochemical gradients of Na and K ions across the plasma membrane. |
| GRIK5 | 19q13.2 | 42502468~42574278 | The protein encoded by this gene belongs to the glutamate-gated ionic channel family. Glutamate functions as the major excitatory neurotransmitter in the central nervous system through activation of ligand-gated ion channels and G protein-coupled membrane receptors. The protein forms functional heteromeric kainate-preferring ionic channels with the related subunits. |
| ZNF574 | 19q13.2 | 42579057~42585719 | Not described |
| POU2F2 | 19q13.2 | 42590262~42636625 | The protein encoded by this gene is a homeobox-containing transcription factor of the POU domain family. The protein binds the octamer sequence 5'-ATTTGCAT-3', a common transcription factor binding site in immunoglobulin gene promoters. |
| DEDD2 | 19q13.2 | 42702745~42724304 | This gene encodes a nuclear-localized protein containing a death effector domain (DED). The encoded protein may regulate the trafficking of caspases and other proteins into the nucleus during death receptor-induced apoptosis. |
| ZNF526 | 19q13.2 | 42724492~42732353 | zinc finger protein |
| GSK3A | 19q13.2 | 42734338~42746736 | Refer to discussion section |
| ERF | 19q13.2 | 42751717~42759309 | Refer to discussion section |
| CIC | 19q13.2 | 42788734~42799949 | This protein encoded by this gene contains a conserved HMG domain that is involved in DNA binding and nuclear localization, and a conserved C-terminus. The N-terminal region of this protein interacts with Atxn1 to form a transcription repressor complex, and in vitro studies suggest that polyglutamine-expansion of ATXN1 may alter the repressor activity of this complex. Mutations in this gene have been associated with [pityriasis versicolor](http://www.malacards.org/card/pityriasis_versicolor) and olidogdendrogliomas. |
| PAFAH1B3 | 19q13.2 | 42801185~42806952 | Refer to discussion section |
| PRR19 | 19q13.2 | 42806284~42814973 | Not described |
| TMEM145 | 19q13.2 | 42817477~42829214 | Not described |
| MEGF8 | 19q13.2 | 42829730~42882921 | The protein encoded by this gene is a single-pass type I membrane protein of unknown function that contains several EGF-like domains, Kelch repeats, and PSI domains. Defects in this gene are a cause of Carpenter syndrome 2, an autosomal recessive genetic condition. |
| CNFN | 19q13.2 | 42891171~42894444 | Not described |
| LIPE | 19q13.2 | 42905664~42931578 | Refer to discussion section |
| CXCL17 | 19q13.2 | 42932695~42947136 | The protein encoded by this gene is a mucosal chemokine that attracts immature dendritic cells and blood monocytes to the lungs. The protein promotes tumorigenesis through an angiogenic activity and also exhibits strong antimicrobial activity. |
| CEACAM1 | 19q13.2 | 43011458~43032661 | The protein encoded by this gene is a member of the carcinoembryonic antigen (CEA) gene family, which belongs to the immunoglobulin superfamily. Diseases associated with CEACAM1 include [microinvasive gastric cancer](http://www.malacards.org/card/microinvasive_gastric_cancer) and [colorectal cancer](http://www.malacards.org/card/colorectal_cancer). |
| CEACAM8 | 19q13.2 | 43084393~43099082 | Not described |
| PSG1~11 genes | 19q13.2~  13.31 | 43225793~43773682 | The human pregnancy-specific glycoproteins |
| PRG1 | 19q13.31 | 43853208~43853700 | Not described |
| CD177 | 19q13.31 | 43857825~43867480 | This gene encodes a glycosyl-phosphatidylinositol (GPI)-linked cell surface glycoprotein that plays a role in neutrophil activation. The protein can bind platelet endothelial cell adhesion molecule-1 and function in neutrophil transmigration. Mutations in this gene are associated with myeloproliferative diseases. |
| TEX101 | 19q13.31 | 43892763~43922767 | Not described |

Note: According to the analysis about all genes involved in this interval, we identify several candidate genes responsible for the complex clinical features observed in our patient, including *RPS19*, *GSK3A*, *ERF*, *PAFAH1B3* and *LIPE* genes. However little is known about functions or clinical significance of other genes in this interval. Therefore the critical region of 19q13.2q13.31 locus is defined.
